# Human mesenchymal stem cells derived from adipose tissue showed a more robust effect than those from the umbilical cord in promoting corneal graft survival by suppressing lymphangiogenesis

**DOI:** 10.1186/s13287-023-03559-2

**Published:** 2023-11-14

**Authors:** Huanmin Kang, Jianing Feng, Yingqian Peng, Yingyi Liu, Yalei Yang, Ying Wu, Jian Huang, Ying Jie, Baihua Chen, Yan He

**Affiliations:** 1 Department of Ophthalmology, The Second Xiangya Hospital, Central South University, Changsha, 410011 Hunan China; 2 Hunan Clinical Research Center of Ophthalmic Disease, Changsha, 410011 Hunan China; 3 Shanxi Eye Hospital, Xi’an People’s Hospital (Xi’an Fourth Hospital), Affiliated People’s Hospital of Northwest University, Xi’an, 710004 China; 4 Department of Obstetrics and Gynecology, The Second Xiangya Hospital, Central South University, Changsha, 410011 Hunan China; 5 Beijing Ophthalmology and Visual Sciences Key Laboratory, Beijing Institute of Ophthalmology, Beijing Tongren Eye Center, Beijing Tongren Hospital, Capital Medical University, Beijing, China

**Keywords:** Mesenchymal stem cells, Corneal transplantation, Allograft rejection, Lymphangiogenesis, Neovascularization

## Abstract

**Background:**

Mesenchymal stem cells (MSCs) have shown promising potential in allograft survival. However, few reports have focused on comparing the immunosuppressive capacity of MSCs from different sources and administered via different routes in inhibiting transplant rejection. Moreover, virtually nothing is known about the role of MSCs in the regulation of graft neovascularization and lymphangiogenesis. In this study, we compared the efficacy of human adipose MSCs (hAD-MSCs) and human umbilical cord MSCs (hUC-MSCs) in vitro and in corneal transplantation models to explore the underlying molecular mechanisms and provide a powerful strategy for future clinical applications.

**Methods:**

hAD-MSCs and hUC-MSCs were generated, and their self-renewal and multi-differentiation abilities were evaluated. The inhibitory effect of human MSCs (hMSCs) was examined by T-cell proliferation assays with or without transwell in vitro. Two MSCs from different sources were separately adoptively transferred in mice corneal transplantation (5 × 10^5^ or 1 × 10^6^/mouse) via topical subconjunctival or intravenous (IV) routes. Allograft survival was evaluated every other day, and angiogenesis and lymphomagenesis were quantitatively analyzed by immunofluorescence staining. The RNA expression profiles of hMSCs were revealed by RNA sequencing (RNA-seq) and verified by quantitative real-time PCR (qRT‒PCR), western blotting or ELISA. The function of the differentially expressed gene FAS was verified by a T-cell apoptosis assay.

**Results:**

hAD-MSCs induced stronger immunosuppression in vitro than hUC-MSCs. The inhibitory effect of hUC-MSCs but not hAD-MSCs was mediated by cell–cell contact-dependent mechanisms. Systemic administration of a lower dose of hAD-MSCs showed better performance in prolonging corneal allograft survival than hUC-MSCs, while subconjunctival administration of hMSCs was safer and further prolonged corneal allograft survival. Both types of hMSCs could inhibit corneal neovascularization, while hAD-MSCs showed greater superiority in suppressing graft lymphangiogenesis. RNA-seq analysis and confirmation experiments revealed the superior performance of hAD-MSCs in allografts based on the lower expression of vascular endothelial growth factor C (VEGF-C) and higher expression of FAS.

**Conclusions:**

The remarkable inhibitory effects on angiogenesis/lymphangiogenesis and immunological transplantation effects support the development of hAD-MSCs as a cell therapy against corneal transplant rejection. Topical administration of hMSCs was a safer and more effective route for application than systemic administration.

**Supplementary Information:**

The online version contains supplementary material available at 10.1186/s13287-023-03559-2.

## Background

Corneal transplantation is one of the most frequent tissue transplantations performed worldwide. However, maximizing graft survival has been a prominent obstacle [1]. Immune rejection remains the leading cause of corneal graft failure [2–4]. Corneal immune-mediated graft rejection is mainly mediated by CD4^+^ T cells, which are stimulated by activated antigen-presenting cells [5]. Newly sprouted lymphatic and blood vessels also contribute to this process by transporting antigen-presenting cells, alloreactive CD4^+^ T cells, and their endogenously released factors (such as INF-γ) [5, 6], resulting in accelerated rejection. Suppressing CD4^+^ T-cell activation and inhibiting angiogenesis are key factors in corneal transplantation therapy [7–9]. Immunosuppressants such as corticosteroids or CsA are the most common treatments to prolong corneal survival time, but their dosage is limited by drug toxicity and life-threatening systemic or blinding ocular complications [10, 11].

Recently, mesenchymal stem cells (MSCs) have been identified as a type of multipotent cell with immunomodulatory and tissue repair potential in various pathological situations [12]. MSCs express low levels of class I and II MHC antigens, which explains their low immunogenicity and makes it possible to widely administer MSCs to normal immunocompetent hosts [13–17]. MSCs can be obtained from many tissues, such as adipose tissue, umbilical cord, and bone marrow [18–20]. MSCs from different sources exhibit tissue-specific characteristics and therapeutic potential [21, 22]. Due to being easily collected from discarded tissues, human adipose MSCs (hAD-MSCs) and umbilical cord MSCs (hUC-MSCs) may be more promising candidates in stem cell-based engineering than those from other sources. hAD-MSCs derived from subcutaneous adipose tissue, are classified as adult stem cells and show increased immunosuppressive potency [22, 23]. The umbilical cord originates from the yolk sac, and the potential of hUC-MSCs is similar to that of embryonic stem cells. hUC-MSCs exhibit rapid proliferation and reduced immunogenic potency even after being exposed to proinflammatory cytokines [24, 25]. Accumulating evidence has indicated the potential of MSCs in alleviating transplantation rejection [26, 27], including corneal transplantation [13, 28–30]. The first human trial of MSCs in corneal transplantation is being conducted [31], which holds promise for stem cell-based tolerogenic therapies (EudraCT number: 2018–000890-60). This trial was designed to adoptively transfer hMSCs to high-risk corneal transplant recipients, such as patients with previous graft failures due to rejection or recipients with abundant blood vessel growth into the graft bed. The application of MSCs is close to clinical translation, but MSC-based therapies still face some important considerations that must be solved before clinical application is feasible. (1) The differences in the abilities of MSCs from different sources to induce immunosuppression and reduce allograft rejection remain unclear. (2) Systemic application of MSCs could lead to embolism and high mortality, which limits their clinical use. Although topical application is possible [13], the strength, weakness and equivalent doses of different administration routes are unclear. (3) The role and mechanism of MSCs in angiogenesis and lymphangiogenesis after transplantation remain unknown.

In this study, we compared the immunosuppressive and therapeutic potential of different doses of hAD-MSCs and hUC-MSCs administered by routes by examining the immunosuppressive functions in vitro and validated the results in an in vivo experimental mouse corneal transplantation model. The results demonstrated that hAD-MSCs showed a stronger ability to suppress graft angiogenesis, lymphangiogenesis and CD4^+^ T-cell proliferation. Compared to systemic application, topical use of hAD-MSCs was more suitable and safer for clinical use based on the lower dose and better efficacy. RNA-seq analysis and validation experiments showed that VEGF-C and FAS are important for the functions of hMSCs in allograft rejection.

## Methods

### Antibodies

All reagent information and concentrations used in this study are listed in Table 1.

**Table 1:** Reagents information and concentration

Antibody and regents	Vendor	Catalog No	Dilution or concentration
*Flow cytometry*			
APC anti-human CD4	Biolegend, California, USA	300,514	1:100
BD Stem flow hMSC analysis Kit	BD Biosciences, California, USA	562,245	According to the manufacture instruction
CellTrace CFSE cell proliferation Kit	Invitrogen, California, USA	C34554	5 μM
Annexin V-FITC/PI apoptosis kit	MULTI SCIENCES, Zhejiang, China	AP 101	According to the manufacture instruction
*Immunofluorescent staining*			
Goat anti-mouse CD31 antibody	R&D Systems, Minnesota, USA	AF3628	1:100
Rabbit anti-mouse LYVE-1 antibody	Abcam Bioscience, Massachusetts, USA	Ab14917	1:100
Alexa Fluor 488-conjugated AffiniPure donkey anti-rabbit IgG (H + L) secondary antibody	Jackson ImmunoResearch Laboratories, Pennsylvania, USA	711–545-152	1:100
Alexa Fluor 594-conjugated donkey anti-goat IgG (H + L) secondary antibody	Jackson ImmunoResearch Laboratories, Pennsylvania, USA	705–585-003	1:100
*hMSC culture medium*			
Basic DMEM/F-12 Medium	Gibco, Shanghai, China	C11330	
Fetal bovine serum	Gibco, Australia	10099141C	10%
Basic fibroblast growth factor	PreproTech, Rocky Hill, USA	100-18B	10 ng/ml
*CD4*^+^ *T cell proliferation culture medium*			
Basic RPMI 1640 Medium	Gibco, Shanghai, China	C11875	
Fetal bovine serum	Gibco, Australia	10099141C	10%
Sodium pyruvate	LIFE technologist, New Mexico, USA	11,360,070	1%
HEPES buffer	LIFE technologist, New Mexico, USA	15,630,080	1%
Nonessential amino acids	LIFE technologist, New Mexico, USA	11,140,050	1%
DYNABEADS HUMAN T-ACT CD3/CD28	Gibco, New York, USA	11161D	According to the manufacture instruction
*Western blotting antibody*			
Thrombospondin-1(D7E5F) Rabbit mAb	Cell Signaling technology, Danvers, USA	#37,879	1:1000
Fas (C18C12) Rabbit mAb	Cell Signaling technology, Danvers, USA	#4233	1:1000
Anti-VEGF-A antibody	Abcam Bioscience, Massachusetts, USA	ab46154	1:1000
Alpha tubulin monoclonal antibody	Proteintech, Chicago, USA	66,031–1-lg	1:10,000
Anti-rabbit IgG, HRP-linked Antibody	Cell Signaling technology, Danvers, USA	#7074	1:2000
Anti-mouse IgG, HRP-linked Antibody	Cell Signaling technology, Danvers, USA	#7076	1:2000
*ELISA*			
Human VEGF-C Quantikine ELISA Kit	Jianglai, Shanghai, China	JL19941	According to the manufacture instruction

### Tissue separation, cell culture, and characterization of hAD-MSCs and hUC-MSCs

Human subdermal adipose tissue was collected from conformed women donors undergoing liposuction cosmetic surgery. The umbilical cord tissue was obtained from healthy pregnant women who were under cesarean section. All donors aged between 18 and 40 years. Ethics approval was obtained from the Ethics Committee of the Second Xiangya Hospital in Central South University (Ethics number: LYF2022048).

The culture of hAD-MSCs was described previously [32]. Briefly, 20 ml human subdermal adipose tissue was digested with equal 1 mg/ml collagenase I (Sigma-Aldrich, Saint Louis, USA) for 2 h at 37 °C with intermittent shaking. The digested adipose tissue was added equal to DMEM/F12 (Gibco, Shanghai, China) containing 10% fetal bovine serum (FBS; Gibco, New Zealand). Then the digested chylomicron-like liquid was filtered by a 70 μm cell strainer and centrifuged at 1500 rpm for 5 min. DMEM/F12 containing 10% FBS was used to wash the cells twice. Finally, the collected cells were suspended with DMEM/F12 containing 10% FBS (Gibco, Australia) and 10 ng/ml basic fibroblast growth factor (bFGF; PreproTech, Connecticut, USA) and seeded in a 75 cm^2^ flask. Regarding the culture of hUC-MSCs, the umbilical cord tissue was first washed with phosphate-buffered saline (PBS) and cut into 2 cm pieces. Then the umbilical vein endothelium, umbilical arteries, and cord adventitia were removed, and Wharton’s jelly was obtained. After minced by scissors, Wharton’s jelly was added equal 2 mg/ml collagenase I and digested at 37 °C for 3 h with shaking. Subsequent steps were the same as described in the culture of hAD-MSCs. After seeding, the culture medium was removed on the third day, and the culture medium was changed every other day. Cells were passaged (1:4) when fusion reach reached 80%. All cells were used in passages 3–5.

hMSCs phenotype was detected by BD Stem flow hMSC Analysis Kit (BD, New Jersey, USA) on the Northern Light Flow Cytometry Instrument (CYTEK NL-3000, USA). Multi-differentiation potentials of hMSCs were performed according to the manufacturer’s instructions and reagents were purchased from Cyagen Oricell (Shanghai, China).

### In vitro PBMC proliferation assay and transwell assays

The immunosuppressive capacity of hMSCs was assessed by their efficacies in inhibiting CD4^+^ T cells. For direct (cell-to-cell contact) coculture, hMSCs were seeded in the 96-well plates overnight before the inoculation of PBMC. hMSCs and PBMC were seeded with different ratios (hMSCs/PBMC = 1:1, 1:2, 1:5, 1:10). A 24-well plate with inserts (0.4 μm pore size) was used to indirect (transwell) coculture. All hMSCs were seeded on the bottom, and PBMC were seeded on top of transwell inserts at a ratio of 1:2 (hMSC/PBMC). PBMC were isolated from heparinized peripheral blood using density gradient centrifugation over Ficoll-Paque (Cytiva, Marlborough, USA), labeled with 5 μM carboxyfluorescein diacetate succinimidyl ester (CFSE; Invitrogen, California, USA) before cocultured with hMSCs. RPMI-1640 (Gibco, Shanghai, China) supplemented with 10% FBS, 1% sodium pyruvate (LIFE technologist, New Mexico, USA), 1% HEPES buffer (LIFE technologist, New Mexico, USA), and 1% nonessential amino acids (LIFE technologist, New Mexico, USA) was used as the co-culture medium. Anti-CD3/CD28 beads (Invitrogen, New York, USA) were used to stimulate T cell proliferation following the manufacturer’s instruction. Positive controls consisted of inoculation of PBMC with anti-CD3/CD28 beads and negative controls consisted of PBMC only. After incubation with hMSCs for 5 days, CD4^+^ T cell proliferation was detected by the Northern Lights Flow Cytometry Instrument.

### RNA-seq analysis

To assess the transcriptome differences between hAD-MSCs and hUC-MSCs in immune modulation, five biological replicates were sequenced for a total of 10 transcriptomes. Cells were dissolved in TRIzol Reagent (Invitrogen, California, USA) following the manufacturer’s instructions and stored at -80 °C till analysis. All mRNA library preparation, quality examination, and RNA sequencing were conducted by Beijing Biomarker Technologies Corporation (Beijing, China). Functional annotation of GO enrichment, KEGG, and clustering analysis was performed using BMK Cloud (www.biocloud.net).

### qRT-PCR analysis

Gene expression in hMSCs was assessed by qRT-PCR analysis. As described above, RNA was extracted from cells using TRIzol Reagent. Complementary DNA (cDNA) was synthesized by PrimeScript RT reagent Kit with gDNA Eraser (TaKaRa, Dalian, China) according to the product manual from the manufacturer. Relative gene expression levels were assessed on a Step One Plus Real-Time PCR System (Applied Biosystems, California, USA) with FastStart Universal SYBR Green Master (Roche, Basel, Switzerland). Relative gene expression levels were normalized to GAPDH and relative expression was calculated using the comparative CT method. All qRT-PCR primers were purchased from Sangon Biotech (Shanghai, China) and listed in Table 2.

**Table 2:** Quantitative real-time PCR primers

Forward Primer	Backward Primer
5′-ATGGGGAAGGTGAAGGTCG-3′	5′-TAAAAGCAG CCCTGGTGACC-3′
5′-CAGCACAACAAATGTGAATGC-3′	5′-GGTTCCCGAAACCCTGAG-3′
5′-GACTCAACAGATGGATTCC-3′	5′-GGGCAGGTTCTTTTACAT-3′
5′-CCCTTCAAAACAAATAGGAGTTCA-3′	5′-ATCCTGTGATTCCAAATGCCAG-3′
5′-CAATGGGGATGAACCAGACTGC-3′	5′-GGCAAAAGAAGAAGACAAAGCC-3′

### Western blotting

Protein expression in hMSCs was detected by western blotting. Total protein in hMSCs was extracted by RIPA lysis buffer (Bester, Shanghai, China) and a bicinchoninic acid kit (Bester, Shanghai, China) was used to determine the protein concentration. A total of 30ug protein in each histone sample per lane was loaded onto a 4–20% gel (Genscript Biotech, Nanjing, China) for western blotting and transferred to a polyvinylidene difluoride (PVDF) membrane (0.22um pore size). Then the PVDF membranes were blocked by 5% non-fat milk at room temperature for 1 h. Following incubating with primary antibodies overnight at 4 ˚C, PVDF membranes were incubated with fluorescein‑conjugated secondary antibodies for 1 h at room temperature. Protein bands were visualized with ProteinSimple FluorChem FC3 System Gel Imager (ProteinSimple, Minneapolis, USA). Image J (NIH Image J system, 1.8.0, Bethesda, USA) was used to quantify protein band intensities with alpha-tubulin (Proteintech, Chicago, USA) as an internal control.

### ELISA

The amounts of secreted VEGF-C were determined by VEGF-C ELISA kits (Bester, Shanghai, China). When 1 × 10^6^ hMSCs were grown to 80% confluence, the supernatants were changed and cell-free supernatants were collected for ELISA analysis according to the manufacturer’s instruction after cells were cultured by 48 h.

### Animals and murine corneal transplantation models

Six to eight-week-old male BALB/c and C57BL/6 mice were purchased from Hunan Slake Jingda Experimental Animals Co. Ltd in China and housed in SPF conditions. As to isogenic transplantation, BALB/c mice were used as both donors and recipients. For the allogeneic transplantation, C57BL/6 mice were used as cornea donors and BALB/c mice served as recipients. Corneal penetrating keratoplasty was performed as previously described [33]. Recipients were anesthetized by intraperitoneal injection of 16.5 mg/ml pentobarbital sodium and a round corneal defect (2.00 mm in diameter) on the right eye was produced. The donor mice were euthanized by cervical dislocation and the full thickness of the central cornea (2.25 mm in diameter) was excised which was continuously sutured in the right eye of the recipient. After the corneal penetrating keratoplasty, hMSCs were administrated randomly according to the design, and tarsorrhaphy was immediately performed with 8–0 needled nylon sutures (Mani, Tochigi, Japan). To minimize potential confounders, the cage of each group was located in sequence. On day 3 post-transplant, the eyelid sutures were removed. The corneal stitches were removed on day 7 post-transplant. Mice of accident death or hyphema were excluded from the study. Then a slit-lamp biomicroscopy system (Leica, Weztlar, Germany) was used to observe and assess all subjects every other day until the endpoint at day 42 (Fig. 2A). The observer was blinded to the experimental group. Grafts’ condition was evaluated by their clarity, edema, and neovascularization. When a graft presents obscured (i.e., invisible pupillary margin and iris texture), it is regarded as rejection.

All animal experiment was performed according to the ethical guidelines for animal and approved by the Experimental Animal Center of Central South University of China (File No. 2022612).

### Administration of hMSCs

hMSCs were filtered through a 70 μm cell strainer and washed with PBS before administration. The dose levels of hMSCs were categorized as low-dose (5 × 10^5^ cells) and high-dose (1 × 10^6^ cells) [29]. Injection of designed hMSCs in 100 μl PBS was included as the experimental group and equal PBS injection as control under a dissecting microscope (Leica, Weztlar, Germany). Subconjunctival administration (SA) was conducted on the right eye (with a graft) while retrobulbar IV injection was performed on the left eye (without a graft) [34]. Before hMSCs administration, the corneal surface was anesthetized with oxybuprocaine hydrochloride eye drops (Santen, Osaka, Japan) 3 times at an interval of 1 min. For subconjunctival injection, lay the needle (30G) against the globe with the bevel parallel to the conjunctiva and insert it into the subconjunctival for 2–3 mm. As to retrobulbar intravenous injection, insert the needle into the medial canthus 2–3 mm (30-degree angle to the nose).

### Histology and quantitative immunofluorescence staining of neovascularization and lymphangiogenesis

Hematoxylin–eosin (H&E) staining was used to evaluate corneal edema and inflammatory cell infiltration. Immunofluorescence was used for quantitative evaluation of corneal neovascularization and lymphatic vessel sprouting. On day 15 post-transplant, the entire eyeball-bearing graft was fixed in 4% formaldehyde (three graft-bearing eyeballs from three random mice for each group). Paraffin-embedded Sects. (5 μm thick) were stained with H&E and mounted on the microscope slides. Immunofluorescence staining and the quantitation of neovascularization and lymphatic perfusion were performed as previously described [33]. To prevent secondary antibody cross-reactivity, lymphatic and blood vessels were performed with sequential double staining. Briefly, the excised cornea (three graft-bearing corneas from three random mice for each group) was permeabilized in acetone for 20 min at room temperature. Corneas were then blocked with PBS containing 10% donkey serum (Absin Bioscience, Shanghai, China) for 1 h. Primary antibody recognizing LYVE-1 (Abcam, Massachusetts, USA) or CD31 (R&D Systems, Minnesota, USA) was, respectively, used to stain corneal lymphatic vessels or blood vessels. Specific secondary antibodies Alexa-Flour 488 (Jackson, Pennsylvania, USA) or 594 (Jackson, Pennsylvania, USA) that correspond to the primary antibody were, respectively, used to incubate corneas for 2 h at room temperature afterward. All antibodies were diluted in 2% bovine serum albumin in PBS. Finally, corneas were mounted with Fluoroshield with DAPI histology mounting medium (Sigma-Aldrich, Saint Louis, USA) and stored with protection from light at 4 °C till imaging. Image J (NIH Image J system, 1.8.0, Bethesda, USA) was used to calculate the percentage of neovessels and neo-lymphatic vessels perfusion area to the area of entire cornea for each mouse.

### CD4^+^ T cells apoptosis assays

The apoptosis rate of CD4^+^ T cells cocultured with hMSCs was evaluated to analyze the role of the FAS/FASL pathway in hMSCs immunomodulate capacity. Anti-FASL (1 μg/ml; R&D Systems, Minnesota, USA) antibody was added to culture system to block FAS/FASL pathway. hMSCs were seeded in the 96-well plates overnight before PBMC inoculation (hMSCs: PBMC = 1:10). Five days later, PBMC in different groups were collected. After anti-human CD4 antibody (BioLegend, California, USA) staining, the Annexin V-FITC/PI apoptosis kit (MULTI SCIENCES, Zhejiang, China) was used to evaluate the apoptosis of PBMC according to the manufacturer’s instruction.

### Statistical analysis

All statistical analyses were conducted by SPSS version 20 (SPSS Inc., Chicago, USA). Corneal graft survival was plotted on Kaplan–Meier survival curves and compared by log-rank tests between indicated groups. The means of two groups were analyzed by unpaired t-test. The significance of multiple experimental groups versus a control group was determined by one-way ANOVA analysis with Dunnett’s multiple comparisons test.

## Results

### Characteristics and differentiation potential of human adipose- and umbilical cord-derived MSCs

**Fig. 1:**
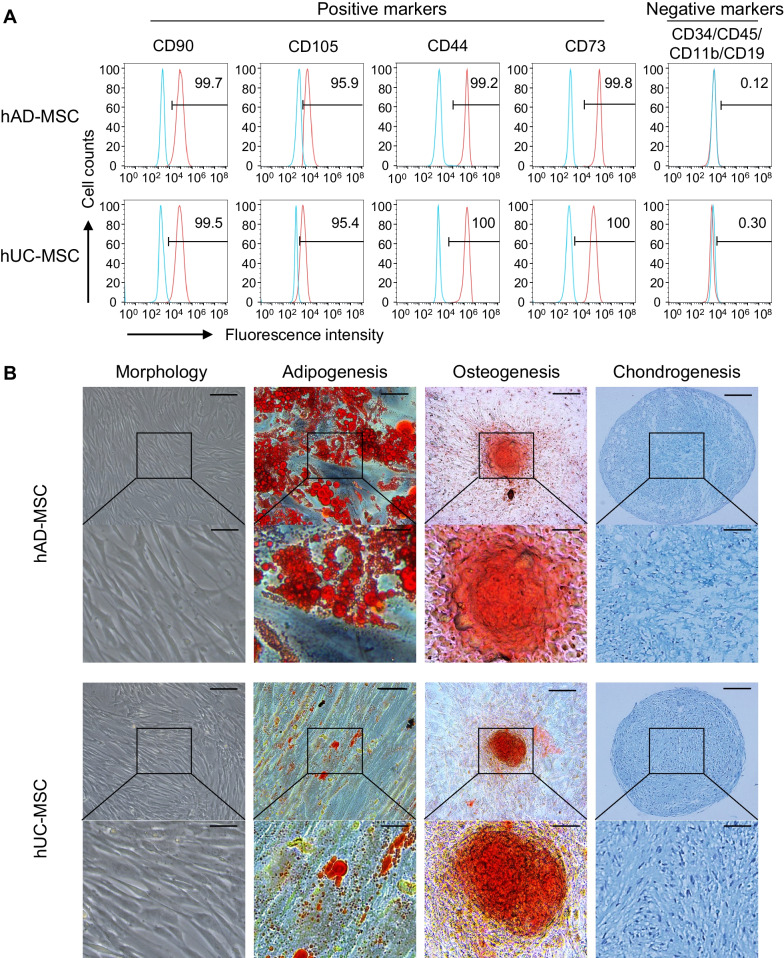
In vitro characterization of hMSCs. **A** Flow cytometry analysis found hMSCs markers CD90, CD105, CD44, CD73 are positive, and CD34, CD45, CD11b, CD19 are negative. **B** Morphology of MSCs and their tri-lineage differentiation. The image showed typical spindle-shaped morphology of adherent hMSCs. Adipogenesis: Triglyceride produced by differentiated adipocytes was stained with Oil Red O. Osteogenesis: Deposited calcium was stained with Alizarin Red. Chondrogenesis: Chondrogenic nodules were stained with Alcian Blue. Scale bar: 150 μm (original), 50 μm (enlarged). Five strains of hAD-MSCs and five strains of hUC-MSCs, respectively, were characterized for the marker expressions and for multi-differentiation potential

MSCs derived from human adipose tissue and umbilical cord were obtained and termed hAD-MSCs and hUC-MSCs, respectively. Both types of MSCs displayed a spindle-like shape and were positive for CD90, CD105, CD44, and CD73 and negative for CD34, CD45, CD11b, and CD19 (Fig. 1A). The differentiation potential of hAD-MSCs and hUC-MSCs was examined, and lipid droplets were much more discernible in hAD-MSCs than in hUC-MSCs. The osteogenic and chondrogenic induction abilities were comparable between hAD-MSCs and hUC-MSCs (Fig. 1B).

### Systemic administration of a lower dose of hAD-MSCs was more effective in prolonging corneal allograft survival than hUC-MSCs

**Fig. 2:**
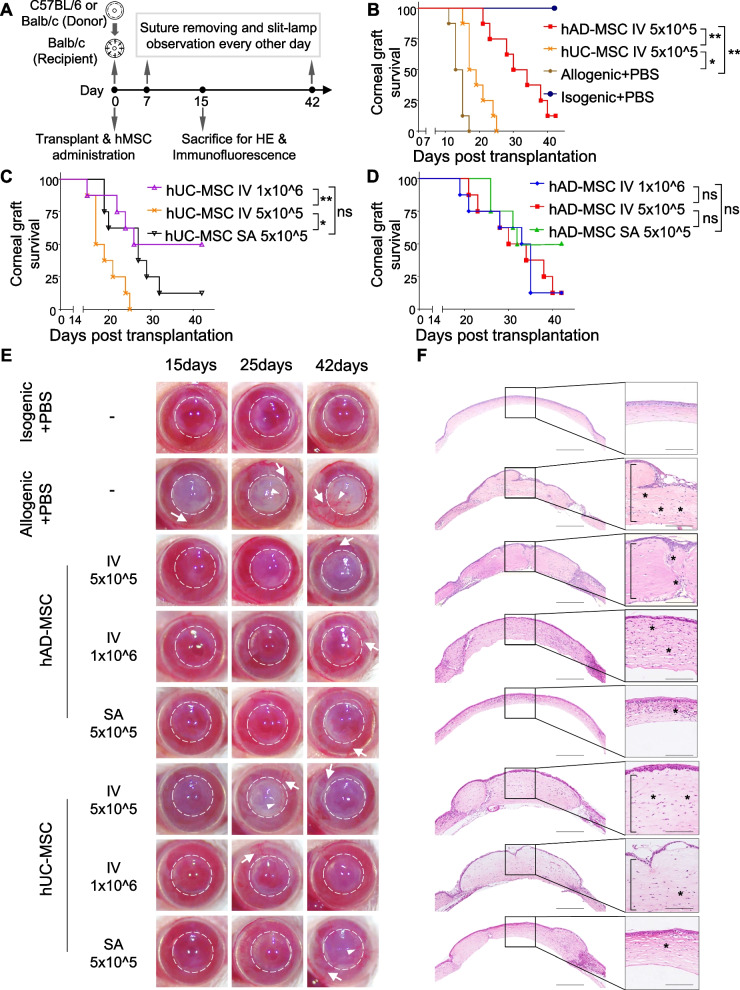
Immunosuppressive function of hMSC in corneal transplantation model. **A** Allogeneic (C57BL/6 to Balb/c) and isogenic (Balb/c to Balb/c) corneal transplantations were performed on day 0 and hMSCs were administrated. Graft-bearing corneas were observed every other day after suture removal. Mice were randomly selected and sacrificed on day 15 and the corneal was obtained for H&E (*n* = 3) and immunofluorescence stain (*n* = 3). **B**: Kaplan–Meier survival curves of isografts and allografts treated with 5 × 10^5^ hMSCs in 100 μl PBS or equal PBS by intravenous (IV) route (*n* = 8 each group). **C** Kaplan–Meier survival curves of allografts with indicated hUC-MSCs by IV or subconjunctival administration (SA). (*n* = 8 each group). **D** Kaplan–Meier survival curves of allografts with indicated hAD-MSCs by IV or SA. (*n* = 8 each group). **E** Representative slim-lamp images (front view) of graft-bearing corneas on day 15, day 25 and day 42 posttransplant. Corneal grafts were outlined by dashed circles. The neovessels in corneal allograft (white arrowheads) and graft bed (white arrows) were presented. **F** H&E stain of graft corneal grafts on day 15 posttransplant from mice treated as in Fig. 2E. The immune cells infiltration (black asterisks) and the areas of stromal edema (black bracket) were presented. IV: intravenous injection; PBS: phosphate buffer saline; *SA* subconjunctival administration. *P < 0.05; **P < 0.01; *ns* not significant. Scale bar: 700 μm (original) and 300 μm (enlarged)

To determine the in vivo therapeutic efficacy of hAD-MSCs and hUC-MSCs in regulating corneal allograft survival, we performed a single systemic administration of 5 × 10^5^ (lower dose) or 1 × 10^6^ (higher dose) hMSCs immediately after corneal transplantation via the IV route (Fig. 2A). Male BALB/c mice were used as recipients for male C57BL/6 donor corneas. Compared to that in the PBS control group, systemic administration of hAD-MSCs and hUC-MSCs resulted in graft prolongation (Fig. 2B). The lower dose (5 × 10^5^) of hAD-MSCs extended the median allograft survival by almost twofold (mean survival time (MST), 32.0 ± 2.6 days, SD, *P* < 0.01, Fig. 2B), significantly reduced neovascularization in the allograft and graft bed (Fig. 2E), and ameliorated epithelial keratinization, immune cell infiltration, stromal edema and collagenous fiber disorder in the allograft (Fig. 2F). In striking contrast, systemic administration of an equivalent number of hUC-MSCs slightly increased median allograft survival (MST, 19.4 ± 1.3 days, SD, *P* < 0.05, Fig. 2B), but there were few effects on reducing allograft neovascularization (Fig. 2E), immune cell infiltration and fiber realignment (Fig. 2F). To further test the effectiveness of hMSCs, a higher dose (1 × 10^6^) of hMSCs was systemically transferred to recipient mice. Interestingly, a significant increase in rejection-free survival was observed in mice treated with the higher dose of hUC-MSCs (MST, 31.9 ± 3.7 days, SD, *P* < 0.01, Fig. 2C), which resulted in the restriction of neovascularization (Fig. 2E) and inflammatory cell infiltration (Fig. 2F). However, the higher dose of hAD-MSCs (1 × 10^6^) failed to further extend allograft survival (MST, 31.0 ± 2.6 days, SD, *P* > 0.05, Fig. 2D). No significant difference in allograft survival was observed between the low- and high-dose hAD-MSCs and high-dose hUC-MSCs groups (*P* > 0.05), which indicated that the effect of hMSCs may plateau. These findings indicate that a lower dose (5 × 10^5^) of hAD-MSCs may be a good option for systemic administration to induce allograft survival, which was more effective than an equivalent or double-dose of hUC-MSCs.

### Topical administration of hMSCs further prolonged corneal allograft survival

Although hMSCs have the advantage of prolonging corneal allograft survival, several limitations reduce the safety of systemic administration, such to the development of pulmonary embolism and reducing the postoperative survival rate [35]. Thus, we performed subconjunctival administration of hMSCs as a topical therapeutic strategy and examined the effectiveness. A single subconjunctival administration of 5 × 10^5^ hMSCs was conducted immediately after corneal transplantation (Fig. 2A). Compared to the equivalent systemic dose of hUC-MSCs, subconjunctival administration resulted in a significant increase in allograft survival (MST, 26.9 ± 3.1 days, SD, *P* < 0.01, Fig. 2C). The MST of the 5 × 10^5^ subconjunctival hAD-MSC group (MST, 35.4 ± 2.5 days, SD, Fig. 2D) was even longer than that of the group that was systemically administered 1 × 10^6^ hAD-MSCs (P > 0.05). Moreover, subconjunctival transfer of 5 × 10^5^ hAD-MSCs and hUC-MSCs reduced graft neovascularization (Fig. 2E) and ameliorated immune cell infiltration and corneal stromal edema (Fig. 2F) to the same extent as 1 × 10^6^ systemically transferred hMSCs. Of note, no mice died after the subconjunctival injection of hMSCs, indicating that subconjunctival administration of MSCs was a much safer and more effective strategy than systemic administration.

### hAD-MSCs were more effective than hUC-MSCs in suppressing corneal graft lymphangiogenesis

**Fig. 3:**
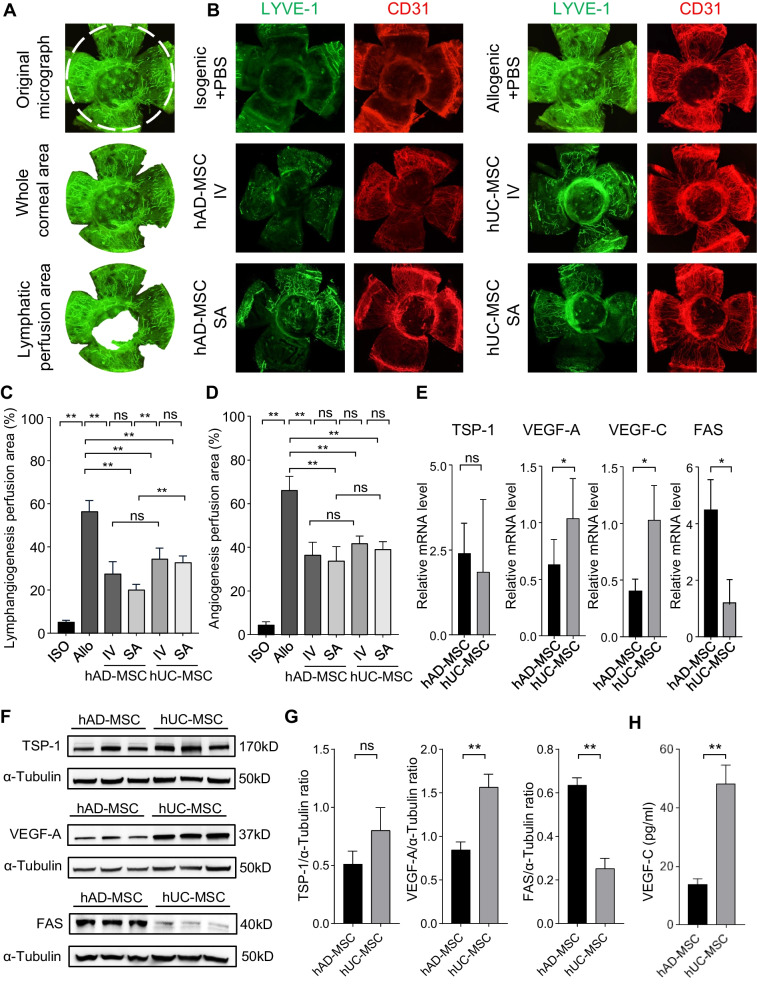
hMSCS alleviate angiogenesis and lymphangiogenesis in the corneal allograft and the graft bed. **A** Image analysis for original immunofluorescent micrograph. The corneal graft was outlined by dashed circle (up). The processed micrographs show the whole corneal area (middle) and lymphatic vessels perfusion area (down). **B** Representative immunofluorescent micrographs for LYVE-1 staining (for lymphatic vessels) and CD31 staining (for blood vessels) of the corneas on day 15 posttransplant in the absence or presence of indicated 5 × 10^5^ hMSCs administration. **C** Quantitation of lymphatic neovessel perfusion area for Fig. 3B (*n* = 3). **D** Quantitation of neovessel perfusion area for Fig. 3B (*n* = 3). **E** The relative mRNA levels of TSP-1, VEGF-A, VEGF-C and FAS in indicate cell were measured by quantitative real-time PCR; all mRNA levels are expressed relative to GAPDH (*n* = 3). **F** The protein expression of TSP-1, VEGF-A, FAS and relevant alpha-tubulin in hMSCs as detected by western blotting. Full-length blots/gels are presented in Additional file 1: Fig. S1. **G** Quantitation of indicated protein expression for Fig. 4F. (H) The protein expression of VEGF-C secreted by hMSCs as detected with ELISA. Bars in (**C**–**E**), (**G**), (**H**) represent mean ± SD of triplicate biological replicates. *P < 0.05; **P < 0.01; *ns* not significant; *P* values were calculated by unpaired *t*-test

To quantitatively evaluate the suppressive effect of hMSCs on corneal graft neovascularization and lymphangiogenesis, immunofluorescence staining of CD31 and LYVE-1 was performed to examine neovessels and lymphatic vessels, respectively. The percentage of the neovessel or lymphatic vessel perfusion area was calculated as previously reported [33], as shown in Fig. 3A. Compared to the PBS control group, systemic and topical administration of hMSCs significantly reduced neovascularization and lymphangiogenesis in the allograft and the graft bed (Fig. 3B–D). Comparison between the groups further revealed that hAD-MSCs showed markedly higher efficacy than hUC-MSCs in suppressing lymphangiogenesis (Fig. 3B,C), which was consistent with the prolongation of graft survival. We then examined the expression levels of the hMSCs-related proangiogenic factors vascular endothelial growth factor A (VEGF-A) and VEGF-C, which are directly related to angiogenesis and lymphangiogenesis, respectively, and the antiangiogenic factor thrombospondin 1 (TSP-1). Compared to hUC-MSCs, hAD-MSCs exhibited less than half of the expression of VEGF-A and VEGF-C and equal levels of TSP-1 (Fig. 3E–H, Fig. 5D). Considering the important role of neovascularization and lymphangiogenesis in corneal graft rejection, these findings indicate that the superiority of hAD-MSCs in prolonging allograft survival may partly rely on their superior inhibitory effect of angiogenesis and lymphangiogenesis.

### hAD-MSCs induced robust immunosuppression in vitro, and the inhibitory effect of hUC-MSCs was mediated by cell‒cell contact-dependent mechanisms

**Fig. 4:**
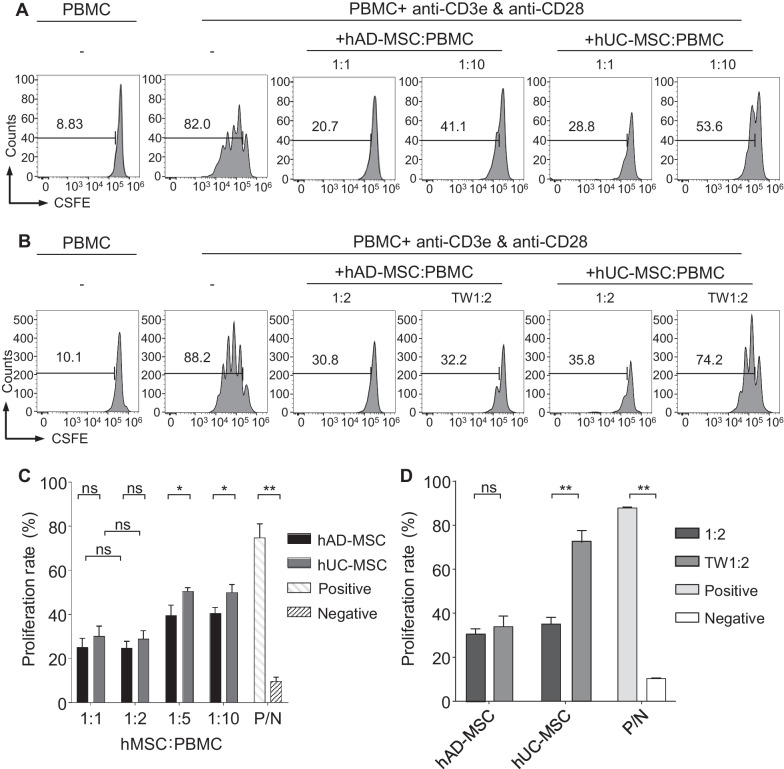
hMSCs exert different immunosuppressive function. **A** Representative flow cytometry histogram shows the proliferation of CFSE-labeled CD4^+^ T cells directly cocultured with hMSCs. hMSCs: PBMC = 1:1 or 1:10. **B** Representative flow cytometry histogram shows the proliferation of CFSE-labeled CD4^+^ T cells directly or indirectly (TW) cocultured with MSCs. MSCs: PBMC = 1:2. **C** Flow cytometric quantitation of CD4^+^ T cells proliferation at different ratios (*n* = 3). **D** Flow cytometric quantitation of CD4^+^ T cells proliferation with or without transwell as shown in Fig. 4B (*n* = 3). Bars in **C** and **D** represent mean ± SD. *N* Negative control that PBMC were left untreated; *PBMC* peripheral blood mononuclear cells; *P* Positive control that PBMC were stimulated by CD3e/CD28 beads; *TW* transwell. All PBMC were stimulated by CD3e/CD28 beads at the ratio of 1:1. *P < 0.05; **P < 0.01; *ns* not significant; *P* values were calculated by unpaired *t*-test

To determine the effect of hMSCs on T-cell proliferation, we cocultured hMSCs with human peripheral blood mononuclear cells (PBMCs) at a gradient ratio and performed a T-cell proliferation assay (Fig. 4A). The results showed that both hUC-MSCs and hAD-MSCs inhibited the proliferation of activated T cells in a dose-dependent manner (Fig. 4A,C). However, the ratio of MSCs: PBMCs = 1:2 resulted in the greatest inhibition, and the extra proportion (1:1) could not further increase the degree of inhibition (Fig. 4C). Furthermore, hAD-MSCs at each proportion exerted greater inhibition than their hUC-MSC counterparts, but the differences in inhibition between MSCs at ratios of 1:1 and 1:2 was not significant (Fig. 4C). These results indicated that the immunosuppressive capacity of hAD-MSCs was approximately 10% higher than that of hUC-MSCs at lower proportions (1:5 and 1:10 ratios), and the capacities of both types of hMSCs reached saturation at higher proportions (1:1 and 1:2 ratios). Next, to investigate the mechanisms of the suppressive effect, we used Transwell (TW)-based coculture assays. Direct contact between MSCs and PBMCs (at a 1:2 ratio) served as the positive control. The results indicated that hUC-MSCs lost their ability to inhibit PBMC proliferation in the TW system, while the inhibitory effect of hAD-MSCs was slightly altered in the TW system (Fig. 4B,D). Saturation of the immunosuppressive activity of hMSCs in vitro paralleled the immunoinhibitory effect in vivo, showing that when the appropriate dose was reached, an increase in the dose of hMSCs could not further prolong graft survival. Moreover, cell‒cell contact played a vital role in the inhibitory effect of hUC-MSCs, validating the finding that topical application of hUC-MSCs is much more effective than systemic administration.

### RNA-seq analysis and confirmation experiments revealed the superior performance of hAD-MSCs in allografts based on reduced expression of VEGF-C and increased expression of FAS

**Fig. 5:**
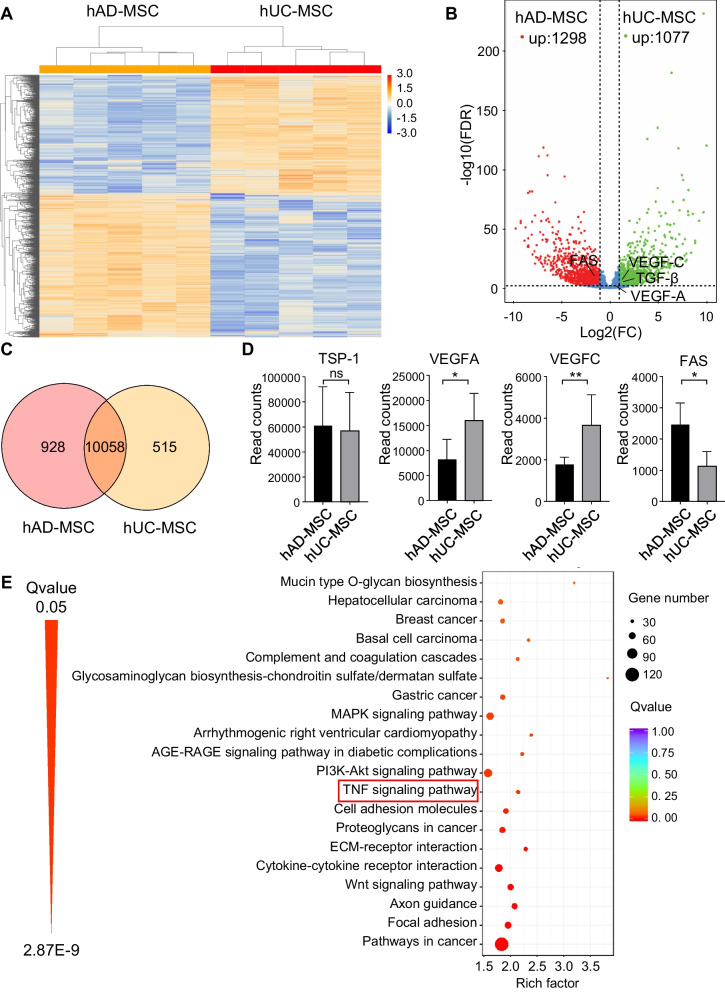
hMSCs RNA sequencing revealed the differential RNA expression in hAD-MSCs and hUC-MSCs **A** Hierarchical clustering of differentially expressed genes in hAD-MSCs and hUC-MSCs group (*n* = 5/group). **B** Volcanic map shows the distribution of differentially expressed genes. The red and green dots indicate significantly upregulated in hAD-MSCs (1298) and hUC-MSCs (1077), respectively (FDR < 0.01 with more than twofold changes). FAS, VEGF-A and VEGF-C were pointed by black lines. **C** Venn diagram indicated the number of genes shared in hAD-MSCs and hUC-MSCs groups. **D** Normalized read counts of TSP-1, VEGF-A, VEGF-C and FAS in hAD-MSCs and hUC-MSCs group (*n* = 5). Bars represent mean ± SD. *P < 0.05; **P < 0.01; *ns* not significant. **E** Top 20 of KEGG pathway enrichment. The circle size represents the number of genes enriched in each indicated pathway. The color of the circle presents the significant *Q* value (corrected *P* value). A lower *Q* value indicates greater pathway enrichment. The Q value of each pathway is in the order of decreasing from upper to lower. FAS related pathway “TNF signaling pathway” were circled by red box

**Fig. 6:**
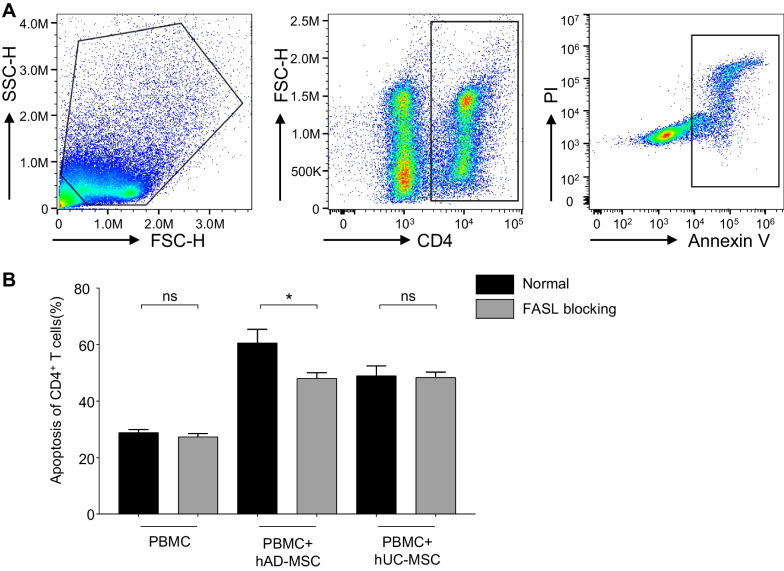
hAD-MSCs induce CD4^+^ T cells apoptosis via FAS/FASL pathway **A** Representative flow cytometry cytograms of CD4^+^ T cells apoptosis after 5 days cocultured with hMSCs. **B** Flow cytometric quantitation of CD4^+^ T cells apoptosis cocultured with hMSCs in the absence or presence of anti-FASL antibody (1 μg/ml). Apoptosis rate was calculated by the sum of early apoptosis and late apoptosis. The bars represent mean ± SD of triplicate biological replicates. *P < 0.05; *ns* not significant; P values were calculated by unpaired *t*-test

To elucidate the regulatory mechanisms and differences between hAD-MSCs and hUC-MSCs in corneal transplantation, whole transcriptome RNA sequencing was performed on hMSCs. Heatmaps showed the clustering of differentially expressed genes (DEGs; fold change > 2 and FDR < 0.01) between hAD-MSCs and hUC-MSCs (Fig. 5A). An overview of DEGs is shown in the volcano map (Fig. 5B). As shown in the volcano map, compared to hUC-MSCs, hAD-MSCs had upregulated expression of 1298 genes, such as ADAMTS8, PRKG2, TBX15, SIX1, IRX2, IRX1 and PLXDC1, and they had downregulated expression for 1077 genes, including DSC3, PCDH10, CHRM2, SPOCK3, ANXA8L1, IL1A and ANXA10. RNA-seq also revealed three differentially expressed genes associated with allograft rejection, including FAS, HLA-F and CD40. Recent studies have suggested that MSCs exert their immunomodulatory effects via the FAS/FASL pathway [36–39]. Our previous study revealed that VEGF-A/C are vital in neovascularization and lymphangiogenesis, which indirectly influence rejection [33]. Thus, we compared FAS, VEGF-A and VEGF-C expression between hAD-MSCs and hUC-MSCs. FAS (−log10 FDR = 7.18, log2FC = −1.11), VEGF-A (−log10 FDR = 1.90, log2FC = 0.95) and VEGF-C (−log10 FDR = 4.02, log2FC = 1.04) are shown by black lines in the volcano map (Fig. 5B). The normalized read counts are shown in Fig. 5D. VEGF-A/C expression was lower while FAS expression was higher in hAD-MSCs, and the results were validated by qRT-PCR and western blot/ELISA at mRNA and protein level (Fig. 3E–H). It should be noted that the reduced VEGF-C expression in hAD-MSCs was consistent with the animal phenotype shown in Fig. 3. The Venn diagram showed overlapping and unique genes between hMSCs (Fig. 5C). The DEGs were mapped to the terms in the KEGG database (Additional file 2: Table S1), and the top 20 pathways were significantly enriched (Fig. 5E). Notably, FAS-associated apoptosis is closely associated with the TNF signaling pathway (ko05206, 32 DEGs, Fig. 5E) [40, 41]. Then, we examined whether differences in the expression of FAS/FASL might affect the immunomodulatory functions of hMSCs. An anti-FASL antibody was added to the coculture system (hMSC: PBMC = 1:10) to block the FAS/FASL pathway. We found that CD4^+^ T-cell apoptosis was decreased when PBMCs were cocultured with hAD-MSCs in the presence of FASL blockade, while hUC-MSCs were hardly affected (Fig. 6A,B). Thus, compared to hUC-MSCs, the FAS/FASL pathway significantly influences the immunomodulatory function of hAD-MSCs by inducing CD4^+^ T-cell apoptosis.

## Discussion

MSCs are a heterogeneous population of immunosuppressive, anti-inflammatory cells and are attractive candidates for stem cell-based engineering. To date, a number of studies have shown that hMSCs are effective in prolonging graft survival, and some human trials have been conducted [13, 29, 42–45]. A study by Fuentes Julian et al. revealed that systemic or local administration of hAD-MSCs did not prolong graft survival in rabbit corneal transplantation but rather increased neovascularization and inflammation [46]. The discrepancies between these studies are probably explained by different experimental conditions, including the cell sources, dose, treatment routes, animal model, injection times and other factors. The standardization of MSC treatment remains the predominant obstacle to clinical application. Many efforts have been made to solve these problems. For instance, MSCs derived from human-induced pluripotent stem cells (iPSC-MSCs) were established and proposed as an alternative to MSCs due to their capacity for indefinite proliferation and mass production [47, 48]. iPSC-MSCs were also shown to be effective in immunomodulation and regeneration, and they have been used to treat many diseases in clinical trials [49–51]. High cost and teratogenesis limit the practical use of iPSC-MSCs [48]. Due to noninvasive collection methods, uncontroversial ethics and large quantities, hAD-MSCs and hUC-MSCs generated from discarded tissue are considered promising candidates [16, 52]. In this study, we performed a side-by-side comparison of hAD-MSCs and hUC-MSCs in a corneal transplantation model. The results showed that hAD-MSCs are better seed cells to prolong corneal graft survival.

Some studies were conducted to analyze the differences among tissue-specific MSCs. HEO et al. compared the immunomodulatory effects of hAD-MSCs and hUC-MSCs by examining in vitro suppression of T-cell proliferation. The study revealed that hAD-MSCs exhibited enhanced immunosuppressive capabilities compared with hUC-MSCs, and the immunosuppressive effects of hMSCs may be associated with HLA-G [22, 53]. Furthermore, other factors, such as IL-10, TGF-β, IL-6, TNF, IL-35 and TSG-6, are related to the immunomodulatory effects of hMSCs [54–58]. These factors (HLA-G, IL-10, TGF-β, IL-6, TNF, IL-35 and TSG-6) were also detected in our study by RNA-seq. The results showed that HLA-G, IL-10, TNF and IL-35 were undetectable in hAD-MSCs and hUC-MSCs. hUC-MSCs expressed higher levels of TGF-β (-log10 FDR = 2.43, log2FC = 1.16) than hAD-MSCs (Fig. 5B; FDR < 0.01 with more than twofold changes). There was no significant difference in IL-6 (−log10 FDR = 0.38, log2FC = 0.83) or TSG-6 (−log10 FDR = 0.02, log2FC = 1.33) expression. The above results were not sufficient to enable reasonable conclusions, which indicated that multiple mechanisms influence the immunosuppressive effects of hMSCs. LI et al. compared the differentiation ability, immunogenicity, proliferation capacity, and suppressive effects of MSCs derived from hAD, hUC, human bone marrow, and human placenta. The results showed that hUC-MSCs performed better than other hMSCs [59]. These reports indicate that the selection of MSCs from an appropriate tissue plays an important role in cell-based therapies. However, all of these reports lack in vivo functional verification, and as far as we know, based on the complex environment, in vitro findings are not always consistent with in vivo effects, especially in different models [42, 60].

Given the risk of complications use of MSCs, a proper dose is crucial to ensure the safety and efficacy of MSC-based therapies. Studies on corneal transplantation typically involved a dose ranging from 2.5 × 10^7^ to 5 × 10^7^ MSCs and 1 × 10^6^ to 1 × 10^7^ MSCs per kilogram body weight in animal models for systemic and topical administration, respectively [46, 61–63]; however, in other solid organ transplantation, a lower dose of 0.26–5 × 10^6^ MSCs was administered in different animal models [42, 64–66]. To the best of our knowledge, there is no consensus about the dose of MSCs used in therapy, and dose selection is empirical. Based on our study, the immunosuppressive effect of hMSCs was dose-dependent within a certain range. Higher doses of hMSCs did not increase the therapeutic efficacy outside this range. This may indicate that the therapeutic efficiency of hMSCs can plateau. A dose of 5 × 10^5^ hAD-MSCs per mouse (2.5 × 10^7^ cells/kg) was shown to be safe and effective in our study.

IV injection was historically performed in most studies. However, IV injection may not be the best MSC delivery route. MSCs that are intravenously administered can be entrapped by lung tissue, which can decrease the therapeutic efficacy and increase the risk of pulmonary embolism [35, 67, 68]. A previous study suggested that intra-arterial transplantation of MSCs was safer than IV administration in an intact porcine model [67]. Another study compared the therapeutic effects of three different delivery routes (IV, intraperitoneal injection, and anal injection) of AD-MSCs on mice with colitis. The results suggested that intraperitoneal injection was the optimum delivery route [69]. Previous studies have also shown that MSC-derived small extracellular vesicles or exosomes are the main effectors and may be promising MSC surrogates based on their safety and versatility [70]. Similar to chemical drug administration, MSC delivery also needs to be selected based on lesion type and the mechanism of MSCs. In our study, subconjunctival topical administration of both hAD-MSCs and hUC-MSCs was safer, easier to perform and more effective than systemic IV administration. Furthermore, after transwell inserts were added, CD4^+^ T-cell proliferation was significantly inhibited by hAD-MSCs but not hUC-MSCs. This finding showed that the immunosuppressive effect of hUC-MSCs was dependent on cell‒cell contact, which may contribute to the improved efficacy of local administration. Compared to other tissues or organs, the cornea is located at the ocular surface, which makes it an ideal model tissue for topical application and the observation of pathological changes and treatment outcomes. This study provides more information and robust evidence supporting future clinical applications.

As a delayed-type hypersensitivity, corneal transplantation failure is mainly caused by underlying inflammation and neovascularization [5, 13]. The original cornea has no blood or lymphatic vessels, and the transfer of activated antigen-presenting cells and alloreactive CD4^+^ T cells strongly relies on the sprouted neo-blood or lymphatic vessels. Studies on a corneal transplantation model demonstrated that the inhibition of neovascularization can prolong corneal graft survival [7, 8]. The VEGF family is closely correlated with neovascularization; VEGF-A and VEGF-C are crucial regulators of angiogenesis and lymphangiogenesis, respectively [71, 72]. In a murine normal-risk corneal transplantation model, VEGF Trap (VEGF-A elimination) administration can decrease neovascularization and improve graft survival [73]. Another study showed that VEGF Trap, anti-VEGF-C and soluble VEGF receptor-3 significantly decreased graft angiogenesis, lymphangiogenesis and lymphoid Th1 cells in a high-risk corneal transplantation mouse model, and all approaches prolonged graft survival; however, VEGF Trap was the most effective in improving long-term graft survival [9]. Decreases in neovascularization and inflammation have also been reported in other ocular surface diseases after MSC administration [74, 75]. L. Espandar et al. reported that hAD-MSCs could reduce corneal vascularization in rabbits and alleviate an ocular alkaline burn model [76]. S. Galindo et al. showed that the development of corneal neovascularization in a rabbit limbal stem cell deficiency model was inhibited by hAD-MSCs transplantation [77]. Corneal graft rejection can be suppressed by decreasing corneal neovascularization, which could also be mediated by MSCs in other ocular surface lesions, but the therapeutic mechanism by which MSCs affect neovascularization in corneal transplantation remains unclear. In our study, angiogenesis and lymphangiogenesis areas in corneal grafts were calculated. hMSCs could reduce angiogenesis and lymphangiogenesis in corneal grafts. Compared to hUC-MSCs, hAD-MSC-related corneal grafts exhibited fewer lymphatic vessels. Then, cornea-related pro/anti-angiogenic factors in MSCs were examined. The RNA-seq results suggested that TSP-1 was comparably expressed in hAD-MSCs and hUC-MSCs, while the prolymphangiogenic factor VEGF-C was reduced in hAD-MSCs. In the presence of a similar level of antiangiogenic factor (TSP-1) expression, the lower level of VEGF-C in hAD-MSCs may partially explain the decrease in lymphatic vessels and longer graft survival time. To the best of our knowledge, this in vitro and in vivo study is the first to show that, compared to that in hUC-MSCs, lower VEGF-C expression in hAD-MSCs reduces corneal graft neolymphangiogenesis and enhances therapeutic efficacy in corneal allograft rejection.

We conducted further studies to investigate the different immunosuppressive capacities of hAD-MSCs and hUC-MSCs. The FAS-FASL pathway in MSCs has been reported to induce T-cell apoptosis and play an important role in immunomodulation [37, 78]. A previous study showed that FAS on mouse BM-MSCs could recruit activated T cells through MCP-1 secretion regulation and subsequently induce immune tolerance in systemic sclerosis and experimental colitis in mice [37]. Another study reported that a microRNA-based strategy was conducive to improving mouse BM-MSC immunotherapy through the FAS/FASL pathway in graft-versus-host disease, inflammatory bowel disease and other immune and inflammatory diseases [39]. The FAS/FASL pathway has rarely been examined in different MSCs, and its role in immunomodulation remains unclear. In our study, qRT-PCR, western blotting and RNA-seq showed that hAD-MSCs expressed higher FAS than hUC-MSCs. In vitro, hAD-MSCs induced a higher CD4^+^ T-cell apoptosis rate than hUC-MSCs. After treatment with anti-FASL, the apoptosis rate of CD4^+^ T cells cocultured with hAD-MSCs was significantly decreased but not in the hUC-MSC group. These results indicate that the increased immunosuppressive effect of hAD-MSCs was dependent on the FAS/FASL pathway.

Although our study helped answer some questions, there are still some important questions about the effectiveness and safety of MSCs. For instance, the efficacy of a second or higher dose and the potential risk of an allogenic response or tumorigenicity require further investigation. Furthermore, VEGF and FAS are both senescence-associated secretory phenotype cytokines, and many articles have shown that the function of MSCs is related to their senescence state [79, 80]. This suggests that the immunosuppressive mechanism of MSCs requires further exploration.

## Conclusions

In conclusion, local hAD-MSCs administration was proven to be an ideal cell-based therapy for corneal allograft rejection based on safety and efficacy. In this study, we compared the different characteristics and immunosuppressive capacities of hAD-MSCs and hUC-MSCs in an in vivo corneal transplant model, an in vitro immunosuppression experiments and by RNA-seq, qRT-PCR, western blotting, and ELISA. Specifically, hAD-MSCs robustly inhibited angiogenesis/lymphangiogenesis and exerted immunosuppressive effects, which were attributed to reduced VEGF-C and increased FAS expression, respectively. Furthermore, local application of hAD-MSCs was proven to be a safer and more effective route of administration than systemic administration. In addition, this is the first study to suggest that VEGF-C and FAS expression in hMSCs is related to corneal allograft rejection and might be a predictive marker of the therapeutic potential of hMSCs. This study contributes to stem cell-based tolerogenic therapies by providing valuable information for selecting the optimal cells and route of administration.

### Supplementary Information

**Additional file 1**: **Fig. S1**. The full-length blots show in Fig. 3F. **A** Original blots for TSP-1 in hAD-MSCs and hUC-MSCs. The boxes outline the protein bands show in Fig. 3F. **B** Original blots for VEGF-A in hAD-MSCs and hUC-MSCs. The boxes outline the protein bands show in Fig. 3F. **C** Original blots for FAS in hAD-MSCs and hUC-MSCs. The boxes outline the protein bands show in Fig. 3F.

**Additional file 2**: **Table S1**. KEGG_enrichment.

## Data Availability

The data generated or analyzed during this study are included in this published article and its supplementary information files. RNA sequencing data in this study are available in the NCBI BioProject database with the BioProject ID PRJNA1033196 (https://www.ncbi.nlm.nih.gov/sra/PRJNA1033196).

## Abbreviations

DEGs: Differently expressed genes
hAD-MSCs: Human adipose mesenchymal stem cells
hMSCs: Human mesenchymal stem cells
hUC-MSCs: Human umbilical cord mesenchymal stem cells
IV: Intravenous
MSCs: Mesenchymal stem cells
MST: Mean survival time
PBMC: Peripheral blood mononuclear cells
PBS: Phosphate buffer saline
RNA-seq: RNA sequencing
qRT-PCR: Quantitative real-time PCR
TSP-1: Thrombospondin 1
TW: Transwell
VEGF-A: Vascular endothelial growth factor A
VEGF-C: Vascular endothelial growth factor C
