# Supplementary figures and images for: Human mesenchymal stem cells derived from adipose tissue showed a more robust effect than those from the umbilical cord in promoting corneal graft survival by suppressing lymphangiogenesis
Source: Stem Cell Res Ther. 2023 Nov 14;14:328. doi: 10.1186/s13287-023-03559-2 (PMC10644560; doi:10.1186/s13287-023-03559-2)

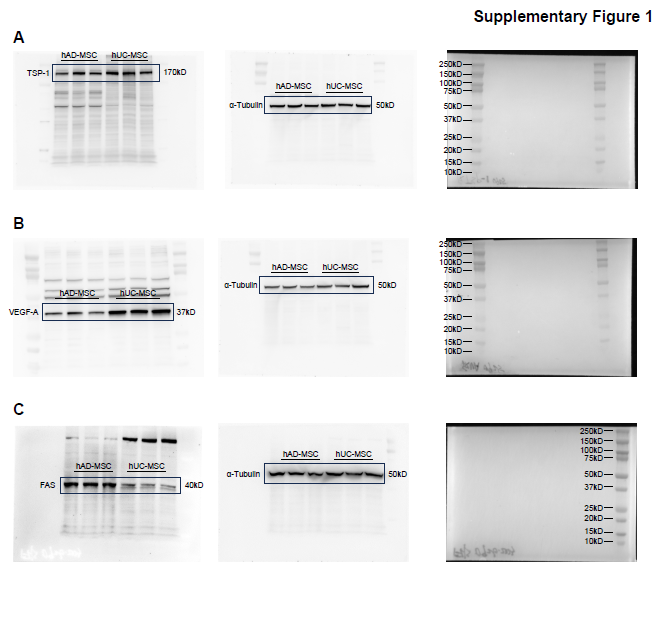

Supplement: Supplementary file 1 — Additional file 1: Fig. S1. The full-length blots show in Fig. 3F. A Original blots for TSP-1 in hAD-MSCs and hUC-MSCs. The boxes outline the protein bands show in Fig. 3F. B Original blots for VEGF-A in hAD-MSCs and hUC-MSCs. The boxes outline the protein bands show in Fig. 3F. C Original blots for FAS in hAD-MSCs and hUC-MSCs. The boxes outline the protein bands show in Fig. 3F. [file 13287_2023_3559_MOESM1_ESM.docx]
